# My Path From Being Mentored to Becoming a Mentor, and Everything in Between

**DOI:** 10.1093/geroni/igaf122.1252

**Published:** 2025-12-31

**Authors:** Lisa Barnes

**Affiliations:** Rush University Medical Center, Chicago, Illinois, United States

## Abstract

Eliminating health disparities and ensuring health equity for all is an important public health imperative. In order to advance knowledge and innovation through research, we must change the healthcare and research workforce so that they are reflective of the communities that face the most health challenges. My goal has always been to be a successful research scientist, and I received excellent training from an institution that was highly ranked in my discipline. But I never received training on being a mentor. Instead, I benefitted from having strong mentoring relationships throughout my academic journey. Relationships that were built on trust, respect, and knowledge-sharing. This presentation will describe my path from being mentored myself to becoming a mentor of others, including students, trainees, post-docs, and junior faculty. I will include insights I’ve learned along the way and offer guidance on how best to support young investigators, especially those under-represented in academia.

